# Experimental investigation of the influence of mine water on mechanical properties of resin grout

**DOI:** 10.1038/s41598-024-65288-7

**Published:** 2024-06-21

**Authors:** Yafei He, Shaowei Liu, Xinxian Zhai, Xiaopeng Li, Biao Hou, Baohua Wang

**Affiliations:** 1 School of Energy Science and Engineering, Henan Polytechnic University, Jiaozuo, 454000 China; 2 Collaborative Innovation Center of Coal Work Safety and Clean High Efficiency Utilization, Jiaozuo, 454000 China; 3 School of Energy and Mining Engineering, China University of Mining and Technology-Beijing, Beijing, 100083 China; 4 Xinzheng Coal Electricity Zhengzhou Coal Industry Group, Zhengzhou, 451184 China

**Keywords:** Resin grout, Mine water solution, Uniaxial compressive strength, Damage parameters, Civil engineering, Coal

## Abstract

Resin grout is widely used in geotechnical and underground engineering, and is often affected by different mine water solutions. This study considered the effects of different mine water solutions and soaking times on resin grout. Soaking tests and uniaxial compression tests were conducted to investigate the changes in the solution pH, relative specimen mass, and uniaxial compressive strength (UCS), and the deterioration of the resin grout’s mechanical properties caused by the mine water solution was analyzed. The corrosion mechanism of resin grout under the action of different mine water solutions was investigated through scanning electron microscopy tests. The results reveal that the pH value of the solution and the relative mass of the specimen gradually stabilized as the soaking time was extended, and the final solution was weakly alkaline. The increase in the acidity and alkalinity of the solution and the extension of the soaking time led to a gradual decrease in the UCS and elastic modulus of resin grout under the action of mine water. As the soaking was prolonged, the resin grout properties deteriorated to different degrees and Poisson’s ratio increased. Moreover, owing to the different types and degrees of mine water action on resin grout in different mine water environments, the changes in the resin grout microstructure were also different. The defined damage parameters can express the damage process of the resin grout’s UCS quantitatively under the action of mine water solution. Finally, beneficial engineering application countermeasures are proposed for different resin grout types used in roadway support applications in coal mines.

## Introduction

**Figure 1:**
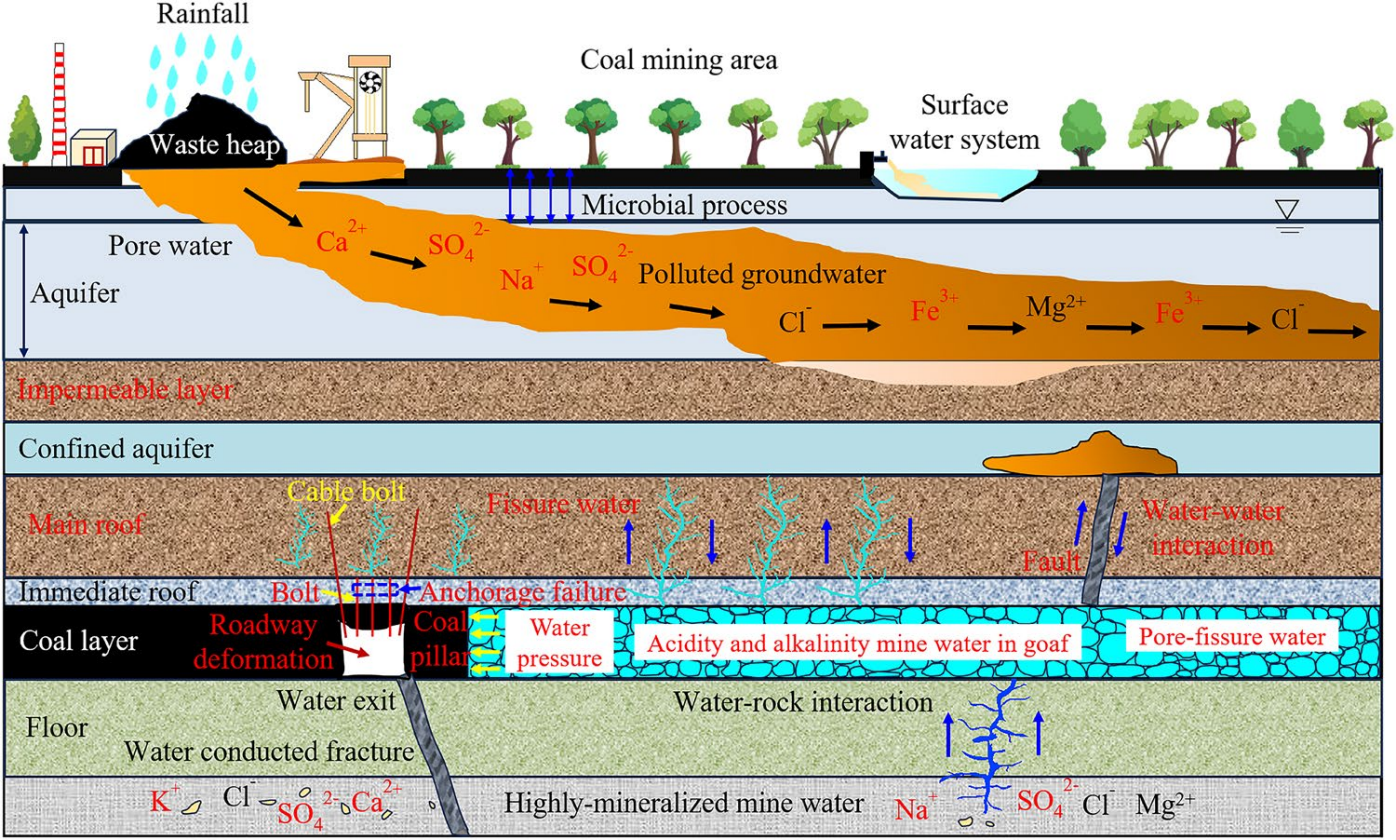
Interactions between coal mine waters and anchoring systems.

As the main type of geotechnical engineering for coal mine roadway support, anchoring has been widely used in China and abroad. In anchoring, resin grout is used as the bonding medium between the bolt/cable bolt and the surrounding rock, and the rock surrounding the roadway and tunnel is reinforced through its interaction with the bolt/cable bolt and resin grout^1,2^. However, owing to the complex and variable geological conditions, the surrounding rock, resin grout, bolt, and surrounding mine water are constantly interacting with various physical, mechanical, and chemical factors. The interaction between the mine water and resin grout in anchoring structures mainly changes the surface characteristics of the structure’s fracture surface through chemical corrosion. Subsequently, the mechanical properties of the anchoring structure deteriorate and the structural strength changes^3–5^. These processes are illustrated in Fig. 1. The resin grout consists of two materials, namely, the resin mastic and the catalyst, and is a polymer composite material. When eroded by mine water solution, the resin grout internal structure and material composition change with time, resulting in the gradual deterioration of the mechanical properties, which poses a severe threat to the long-term stability of the anchoring system^2,6–8^. Therefore, investigating and analyzing the corrosion effects of mine water on the mechanical properties, such as the strength and deformation of resin grout, has important theoretical and engineering value.

In recent years, numerous studies, both in China and abroad, have extensively investigated the effect of corrosion caused by mine water solution on the mechanical properties of the bolt/cable bolt and surrounding rock. Chu et al.^9,10^ simulated the corrosion rate of the bolt/cable bolt in mine water, and observed that different bolt/cable bolt types had different corrosion rates in different mines. The corrosion rate was highest under strong acidic conditions, and slightly smaller under neutral and alkaline conditions. Spearing et al.^11,12^ and Wu et al.^13^ investigated the corrosion status of bolts in underground coal mines, and reported that the stress corrosion of bolts is an important problem related to the premature failure of bolts in underground coal mines. Yu et al.^14^, Sun et al.^15^, and Peng et al.^16^ investigated the mechanical properties and microscopic characteristics of rock in different hydrochemical environments and obtained the corresponding variation laws. Wu et al.^17^ investigated the physical and mechanical properties of mortar specimens in different water chemical solutions and different corrosion cycles, and reported that different water chemical solutions have different effects on strength. Moreover, a new damage parameter was defined based on the mechanical properties after chemical corrosion, and the degree of corrosion degradation of mortar specimens by a chemical solution was indirectly analyzed.

The influence of mine water solution on the mechanical properties of resin grout in anchorage structures has been partially investigated. German scholars soaked pure resin test blocks in seawater for 15 months, subjecting them to weathering, and found that blocks with different strengths had different retention rates^18^. The mechanical properties, such as the compressive strength, tensile strength, and cohesive force of unsaturated polyester resin grout, have been measured at different temperatures by the China General Research Institute of Mining and Metallurgy. The results revealed that the compressive strength and tensile strength of the resin decreased rapidly as the temperature increased^19^. Zheng and Huang^18^ investigated the strength of resin grout under different conditions by conducting a boiling-water accelerated test and a soaking water test at room temperature, and found that the mechanical properties and surface stability of the resin grout prepared from different resin varieties were very different under identical conditions. Gao et al.^20^, Zhou and Zhou^21^, and Zheng et al.^22^ investigated the water and acid–base corrosion resistance of unsaturated polyester resin with different chemical structures and concluded that the corrosion resistance of resin is related to the resin’s molecular structure, and that temperature has a certain effect on the corrosion resistance of resin. Aziz et al.^23–25^ tested various resin grout properties and proposed a new method for preparing resin grout specimens. Li^26^ investigated the change law of the compressive strength of the resin grout in an alkaline water environment, and concluded that the compressive strength of resin grout in different alkaline water environments and different corrosion cycles exhibits a decreasing trend compared with the natural state. As the alkalinity of the solution increases, the degradation of the compressive strength of resin grout becomes more severe. However, systematic experimental studies on the mechanical properties and corrosion mechanisms of resin grout after mine water corrosion are rare. Owing to the deterioration of the physical and mechanical properties of resin grout under the action of mine water solution, the strength characteristics and stability of the anchoring system are severely affected. Therefore, it is necessary to investigate the degradation of the mechanical properties and the corrosion mechanism of resin grout after corrosion in mine water environments.

This study considered the structural change of resin grout in mine water solution as the breakthrough point, and analyzed the influence of the mine water solution’s pH value on the specimens’ physical and mechanical properties. The pH value, relative mass, and porosity change rate of specimens were monitored during the process of corrosion by the mine water solution. The variation with time of the mechanical properties of the specimens in the mine water solution was investigated, and the chemical corrosion mechanism of the specimens under the action of mine water is discussed. The findings of this study provide a useful reference and guidance for the improvement of resin grout applications.

## Test materials, equipment, and methods

### Specimen preparation

In this study, the investigated resin grout specimens were prepared according to the relevant resin grout standards (MT 146.1-2011)^27^. The resin grout mainly consisted of resin, filler, catalyst, and accelerator. The resin was unsaturated polyester resin, the filler was dolomite powder, and the catalyst contained benzoyl peroxide, light calcium, and water^28^.

Bulk slow-speed resin grout was used to prepare the specimens, and a catalyst with a gel time of 180 s was used. The catalyst amount was 5% of the resin mastic base^2^. The resin mastic and catalyst used in the test were weighed using an electronic scale with an accuracy of 0.01 g. The weighed resin mastic and catalyst were poured into the mixing barrel in turn and quickly and evenly stirred to carry out the corresponding molding. The molding process was conducted in strict accordance with MT146.1-2011. The uniformly stirred mixed resin grout was quickly filled into a mold that was pre-placed on the vibrator. The mold size was 400 mm × 400 mm × 400 mm. The mold was vibrated by the vibrator and manually compacted before being scraped flat and left standing for a few minutes. When the resin grout had completely cured and reached a certain strength, it was removed from the mold. Then, a cylinder with a diameter of 50 mm and length of 100 mm was drilled from the processed specimen. To ensure the uniformity of the specimens and the comparability of the test data, and reduce human error as much as possible, the drilling rig speed and mixing time were kept as equal as possible in the specimen fabrication process.

**Figure 2:**
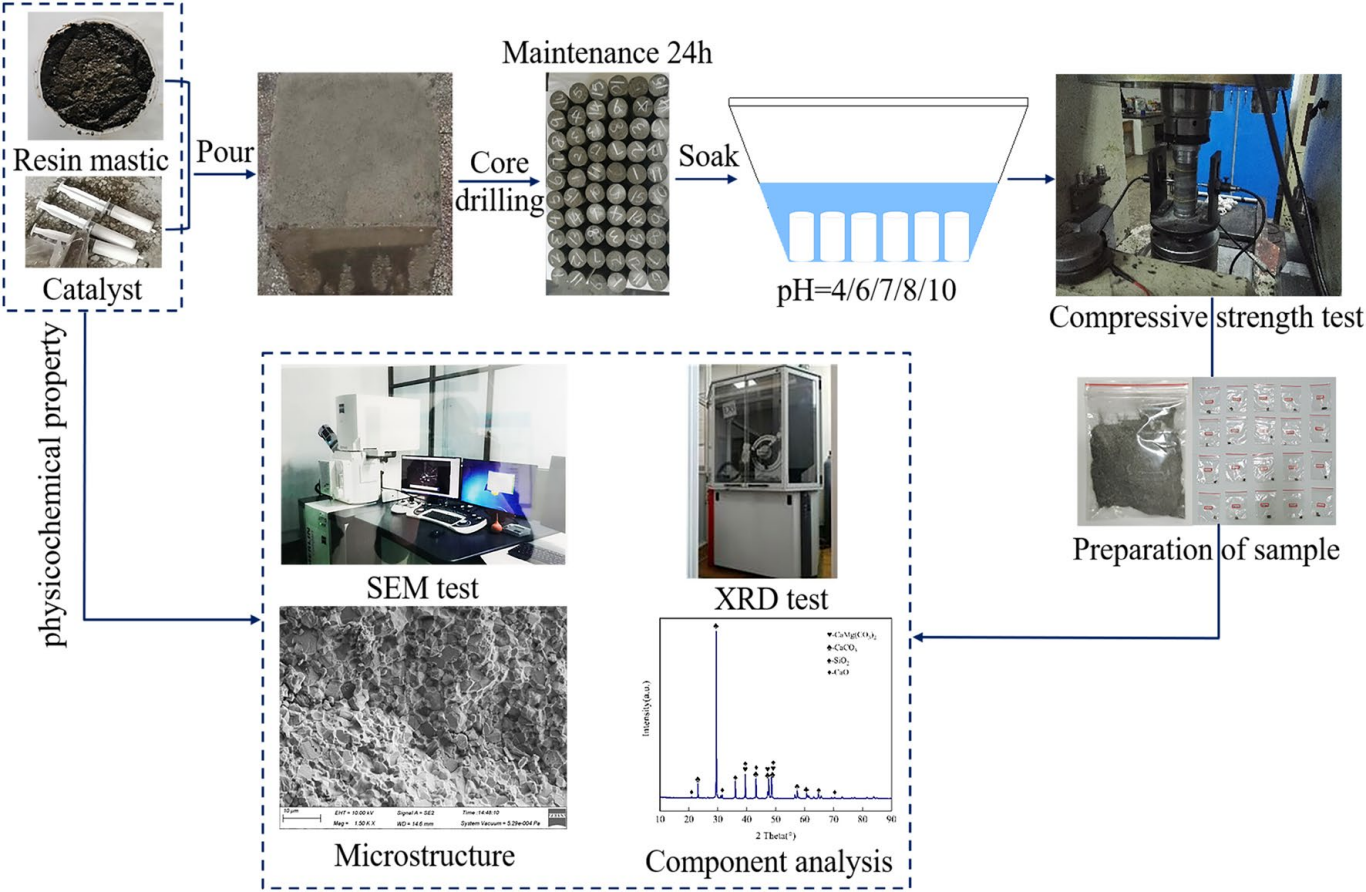
Test flow chart.

According to GB/T 23,561.7-2009 “Methods for determining the physical and mechanical properties of coal and rock. Part 7: Methods for determining the uniaxial compressive strength (the uniaxial compressive strength denoted as UCS or *R*_c_) and counting softening coefficient”^29^, the two ends of the specimen after demolding were polished such that the non-parallelism of the two ends did not exceed 0.05 mm, the flatness of the end face did not exceed 0.02 mm, and the axial deviation did not exceed 0.25° to ensure adequate surface smoothness and avoid errors caused by an irregular surface. The test process is shown in Fig. 2.

### Test equipment and parameters

A standard resin grout specimen was subjected to a uniaxial compression test on an RMT-150b electro-hydraulic servo rock testing machine. The pH value of the mine water solution was measured using a pH meter. The measurement range of the instrument was 0.00–14.00, and the error range was ± 0.05. The mass of the specimen was weighed using an electronic scale with an accuracy of 0.01 g. The true density of the specimens was measured using a 3H-2000TD1 true density analyzer.

### Preparation of mine water solution

The mine water in roadways and tunnels is a very complex chemical solution, and mainly consists of the groundwater, atmospheric precipitation, surface water, and goaf water found in the ore body and surrounding rock voids^30^. Under the influence of the geological age, geological structure, mineral composition of coal measures, and other factors, the mine water composition changes with time. The pH value varies greatly in different environments. The mine water solution mainly contains cations such as Na^+^, Ca^2+^, Mg^2+^, and K^+^, and anions such as Cl^−^, SO_4_^2-^, and HCO_3_^-^. The experimental process cannot consider the effect of all anions and cations on the performance of resin grout. Therefore, Na^+^ and Cl^−^, which are relatively abundant in groundwater, were selected as the electrolyte solutes for the mine water solution used in this study^31^. During the test, the influence of the acidity and alkalinity of the mine water solution on the resin grout performance was mainly considered, and NaCl solutions with different pH values were prepared. By increasing the concentration of the solution, the effects of the long-term interaction between the mine water solution and the resin grout can be reflected more accurately within a shorter time.

Studies from both China and abroad were reviewed and used as guidance to realistically simulate the complex mine water environment wherein the anchorage structure is located. On the basis of the literature and results obtained by previous studies, the pH value of mine water in many mining areas in Shanxi, Inner Mongolia, and Xinjiang is 2.63–9.75^26,30,32,33^. Therefore, five electrolyte solutions with the pH values of 4, 6, 7, 8, and 10 were prepared, denoted as A, B, C, D, and E, respectively. The electrolyte was 0.1 mol/L NaCl. The prepared mine water solutions are listed in Table 1. The pH value of the acidic solution was adjusted by adding 36–38% HCl, and the pH value of the alkaline solution was adjusted by adding 96% NaOH solid particles. The specific preparation process of the solution is as follows. First, NaCl was added to distilled water and fully stirred with a glass rod such that it could completely dissolve; second, a certain amount of 95% HCl solution or NaOH solution was pipetted. Then, the reagent was dropped into the NaCl solution and stirred evenly with a glass rod. Finally, the pH value of the solution was monitored using a pH meter until the pH value of the solution reached the set range.

**Table 1:** Prepared mine water solutions.

Solution designation	Solution type	Electrolyte	Concentration (mol/L)	pH value
A	Acidic	NaCl	0.1	4
B	Acidic	NaCl	0.1	6
C	Neutral	NaCl	0.1	7
D	Alkaline	NaCl	0.1	8
E	Alkaline	NaCl	0.1	10

### Test scheme

According to the test requirements, six groups of test specimens, with three specimens in each group, were processed. One group of specimens, that is, the group of specimens in the natural state (NS), was not soaked in the mine water solution, whereas the other five specimen groups were treated with mine water solution, and all groups were soaked in different solutions for different time periods during the test. First, the processed specimens were numbered one by one. Second, the initial mass, diameter, and height of each specimen were measured, and the specimens were photographed. Then, the numbered standard specimens were put into 5 L of five different types of mine water solution, which was prepared in advance for soaking. During the soaking process, the solution was kept closed to prevent contact with external ions or impurities that would affect the test results. The solution pH value, specimen mass, and porosity change were measured regularly. The measurement time was determined according to the change of the solution pH value, and the measurement continued until the solution pH value tended toward stability for a long time, which indicated that the interaction between the specimen and the mine water solution had reached saturation. At this time, the measurement of the corresponding pH value and specimen mass was discontinued.

The test process consisted of three cycles: soaking for 10 d, 20 d, and 35 d. When a cycle was completed, the specimen was removed from the mine water solution and wiped clean with cotton cloth in preparation for the uniaxial compression test. Finally, the resin grout was observed using scanning electron microscopy (SEM). The soaking scheme for the resin grout specimens in different mine water environments is presented in Table 2.

**Table 2:** Resin grout specimen soaking scheme under different mine water environments.

Solution type	Samples	Electrolyte	Concentration (mol/L)	Soaking time (d)
NS	NS-1	–	–	–
	NS-2			
	NS-3			
pH = 4	10-1	NaCl	0.1	10
	10-2			
	10-3			
	20-1	NaCl	0.1	20
	20-2			
	20-3			
	35-1	NaCl	0.1	35
	35-2			
	35-3			
pH = 6	10-1	NaCl	0.1	10
	10-2			
	10-3			
	20-1	NaCl	0.1	20
	20-2			
	20-3			
	35-1	NaCl	0.1	35
	35-2			
	35-3			
pH = 7	10-1	NaCl	0.1	10
	10-2			
	10-3			
	20-1	NaCl	0.1	20
	20-2			
	20-3			
	35-1	NaCl	0.1	35
	35-2			
	35-3			
pH = 8	10-1	NaCl	0.1	10
	10-2			
	10-3			
	20-1	NaCl	0.1	20
	20-2			
	20-3			
	35-1	NaCl	0.1	35
	35-2			
	35-3			
pH = 10	10-1	NaCl	0.1	10
	10-2			
	10-3			
	20-1	NaCl	0.1	20
	20-2			
	20-3			
	35-1	NaCl	0.1	35
	35-2			
	35-3			

## Mine water solution soaking test and analysis of results

### Variation of pH value of mine water solution

**Figure 3:**
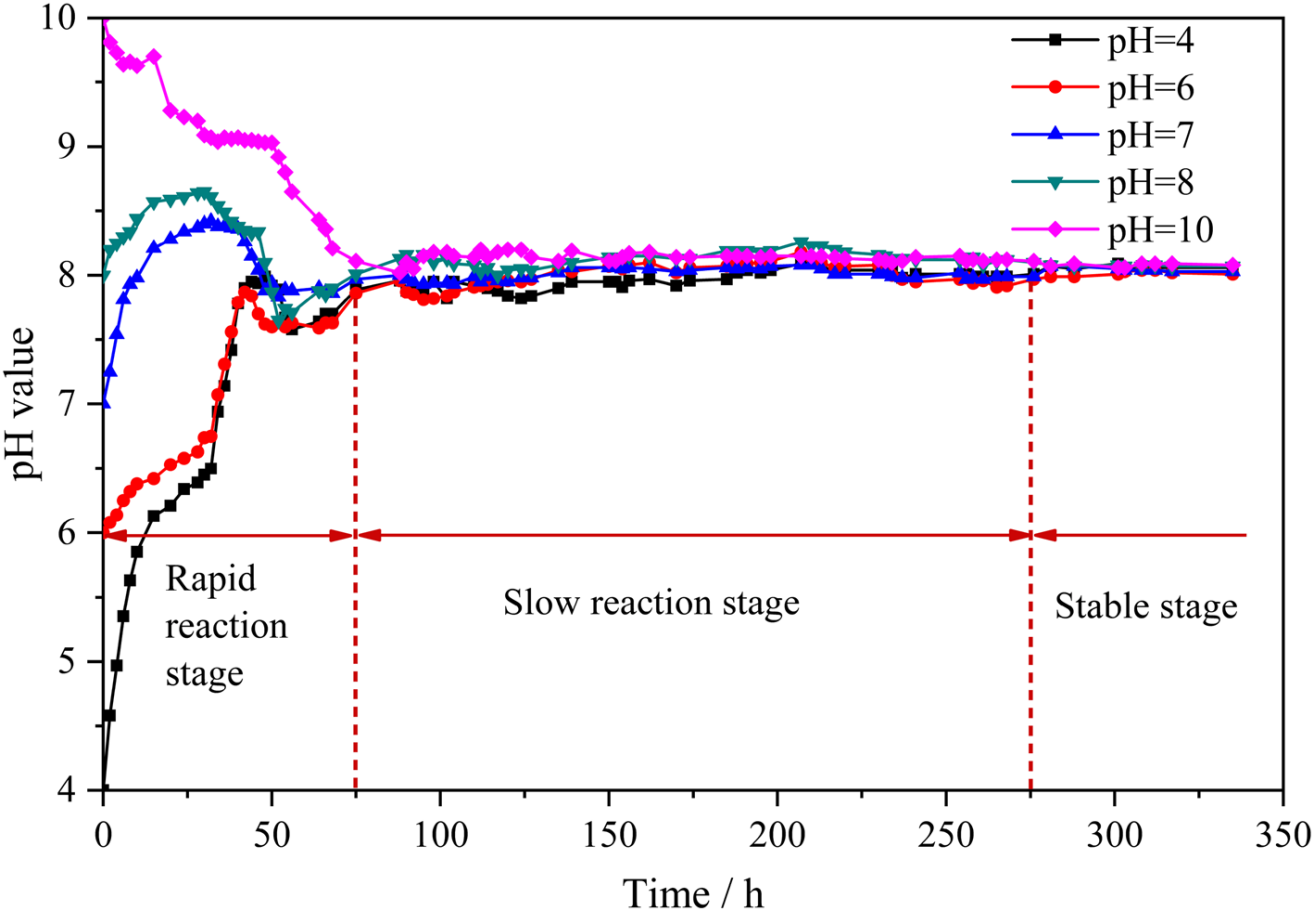
Relationship between pH value of mine water solution and time.

The change in the pH value of the mine water solution was measured using a pH meter. The measurement time started from the moment when the specimen was completely soaked in the mine water solution, and the pH values of various mine water solutions were measured sequentially. The measurement time interval was determined based on the rate of change in the pH value of the solution to obtain the variation law of the pH values of various mine water solutions with time, as shown in Fig. 3.

In Fig. 3, the following observations can be made:

1. Regardless of the initial acidity and alkalinity of the mine water solution, the pH value of various mine water solutions gradually tended toward weak alkalinity over time. The main reason for this is that the H^+^ ions in the solution gradually decreased under the action of chemical corrosion. The resin grout fillers, specifically dolomite powder (mainly CaMg(CO_3_)_2_) and light calcium, underwent chemical reaction or a small amount of hydrolysis under both acidic and alkaline conditions, resulting in a weakly alkaline solution. Moreover, the ester bond of polyester resin in unsaturated polyester resin was particularly susceptible to hydrolysis by acid and alkali, and exhibited weak alkalinity after hydrolysis^34^.
2. Specimens soaked in mine water solution interacted with the solution. On the basis of the amplitude of the pH change of the mine water solution, the entire process of interaction can be divided into three approximate stages. During the rapid reaction stage (0–75 h), the pH value of each mine water solution changes in an obvious manner. In the slow reaction stage (75–275 h), the pH value of the water solution of each mine changes slightly. In the stable stage (after 275 h), the pH value of each mine water solution remains essentially constant. In the pH 4, 6, 7, 8, solutions the pH value first increased, then decreased, and then the change range gradually narrowed until a stable range was eventually reached. The pH value in the pH 10 solution first decreased, then the change in the pH value gradually became smaller, and a stable range was finally reached. This indicates that the interaction between the specimen and the mine water solution has strong time dependence.
3. When the specimen was soaked in acidic solution, the pH value increase was greater than that when the specimen was soaked in alkaline solution. The main reason for this is the chemical interaction between the internal substances of the resin grout and acid under acidic conditions, which led to a drastic change in the solution pH value. Under alkaline conditions, it is not easy for the resin grout internal substances to react with the alkaline solution, but the ester bond in the cross-linked network of the unsaturated polyester resin is easily hydrolyzed; therefore, the solution pH value does not change much.

### Change of porosity of specimens before and after soaking

Under the action of different mine water solutions, some mineral components on the surface and even inside the resin grout interact with the mine water solution, which results in the formation of voids and cracks. This increases the porosity of the original material, and thus affects the resin grout mechanical properties. The effect of the mine water solution on the void structure of the resin grout depends on time, and the porosity of the specimen varies with different corrosion cycles, as follows^4^:

1 $$ n = \left( {1 - \frac{{\rho_{g} }}{\rho }} \right) \times 100\% $$

where *n* is the total porosity of the specimen; *ρ*_g_ is the dry apparent density of the specimen; *ρ* is the true density of the specimen.

This study defined a physical quantity that can reflect the degree of void change in the resin grout, that is, the porosity change rate *η*, which is expressed as follows:

2 $$ \eta = \frac{{n_{t} - n_{0} }}{{n_{0} }} $$

where *n*_t_ is the porosity of the specimen after corrosion, and *n*_0_ is the porosity of the specimen before corrosion.

**Figure 4:**
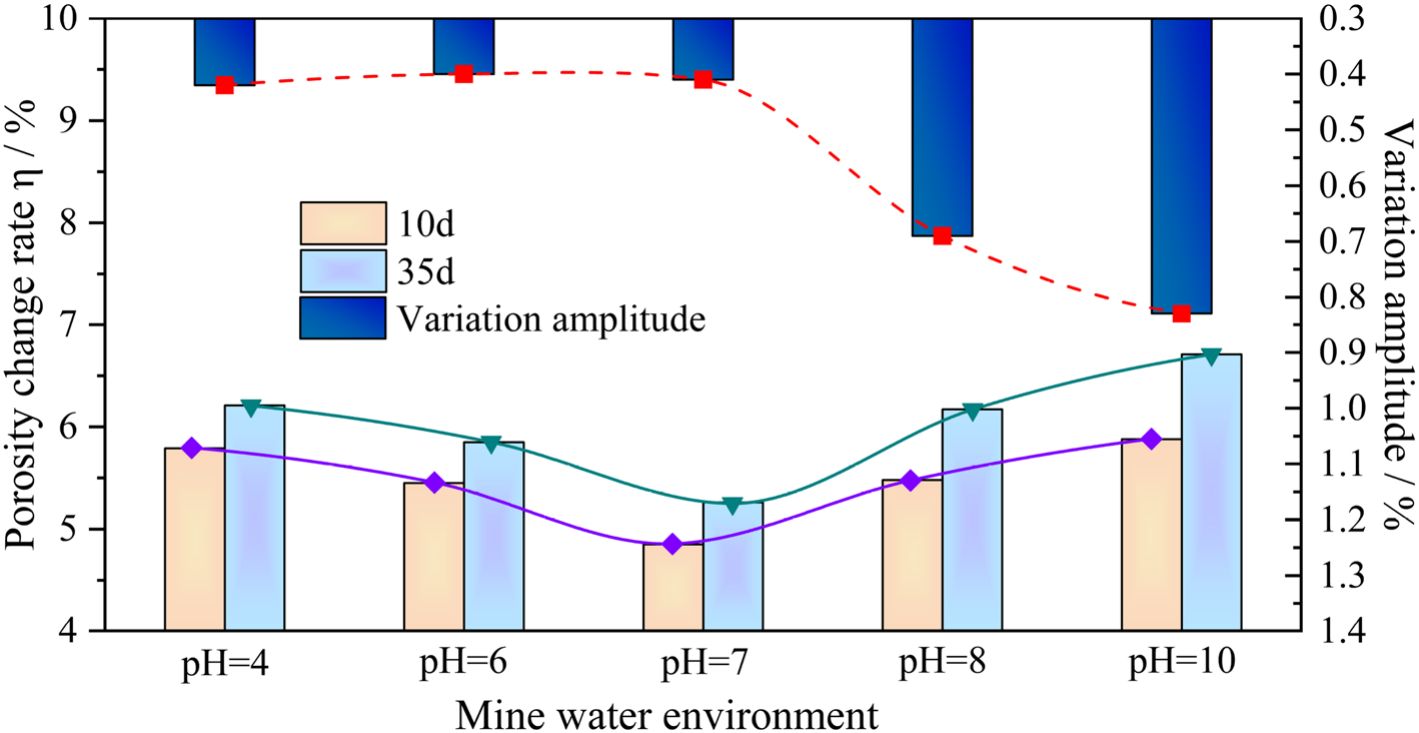
Relationship between porosity change rate and pH value of specimens soaked for 10 d and 35 d.

Owing to similar changes in the porosity of resin grout specimens in different mine water environments, specimens from the two test cycles of 10 d and 35 d were selected in this study. The porosity and porosity change rate of the resin grout specimens before and after corrosion under different mine water solution conditions were statistically analyzed, and the results are presented in Table 3. Figure 4 shows the curve of the porosity change rate of the resin grout specimen with the solution pH value after soaking for 10 d and 35 d, respectively.

**Table 3:** Comparison of porosity change rate of resin grout specimens before and after corrosion in mine water environment.

Chemical solution	Soaking time (d)	pH value	Concentration (mol/L)	Original porosity (%)	Porosity after corrosion (%)	Porosity change rate (%)
NaCl	10	4	0.1	6.73	7.12	5.79
NaCl	35	4	0.1	6.76	7.18	6.21
NaCl	10	6	0.1	6.79	7.16	5.45
NaCl	35	6	0.1	6.84	7.24	5.85
NaCl	10	7	0.1	6.81	7.14	4.85
NaCl	35	7	0.1	6.85	7.21	5.26
NaCl	10	8	0.1	6.94	7.32	5.48
NaCl	35	8	0.1	6.97	7.40	6.17
NaCl	10	10	0.1	6.97	7.38	5.88
NaCl	35	10	0.1	7.00	7.47	6.71

From Table 3 and Fig. 4, the following conclusions can be drawn:

1. The porosity of the resin grout specimen after corrosion was significantly higher than that before corrosion, indicating that the mine water solution interacted with the resin grout specimen, which caused the formation of voids and cracks and resulted in the higher porosity of the specimen. The porosity change rate of the specimen increased with the chemical corrosion time.
2. When the resin grout specimen was soaked in an acidic or alkaline solution under different pH value conditions for the same time period, as the acidity or alkalinity of the solution became stronger, the porosity change rate of the resin grout specimen became greater, and the two are positively correlated. With the same pH value and different time periods, when the resin grout specimens were soaked in different solutions for 35 d, the relative variation amplitudes compared with 10 d were 0.42%, 0.4%, 0.41%, 0.69%, and 0.83%, respectively. Moreover, as the test period increased, the porosity change rate of the resin grout specimen became greater, and the two were positively correlated. This shows that the porosity change rate of the resin grout specimen is directly related to the strength of the solution’s acidity or alkalinity, and the test period.
3. With the other conditions being the same, the influence of various mine water solutions on the porosity change rate of the specimen is as follows: the porosity change rate in an acidic or alkaline solution is greater than that in a neutral solution.

### Relative mass change of specimens before and after soaking

The analysis and comparison of the quality of the specimens before and after soaking can indirectly reflect the interaction process between the resin grout specimen and the mine water solution. The specific method is as follows: Before putting the finished resin grout specimen into the mine water solution, the original mass was weighed as *m*_0_, and then soaked. During the soaking process, when the specimen reached the time specified in the test, it was removed from the mine water solution, and the mine water solution on the surface of the specimen was wiped off with a clean cotton cloth such that there was no residual liquid on the surface of the specimen. At the same time, the wiped specimen was allowed to stand in air for 10 min to ensure that the mine water liquid on the surface of the specimen had completely volatilized and dried. Finally, an electronic scale was used to weigh its mass as *m*_i_. According to the mass of the specimen before and after the measurement, the relative mass calculation formula of the specimen is expressed as follows:

3 $$ \Delta m = m_{i} - m_{0} $$

where *m*_0_ and *m*_i_ denote the quality of the specimen before and after corrosion, respectively; Δ*m* is the relative mass of the specimen before and after corrosion.

**Figure 5:**
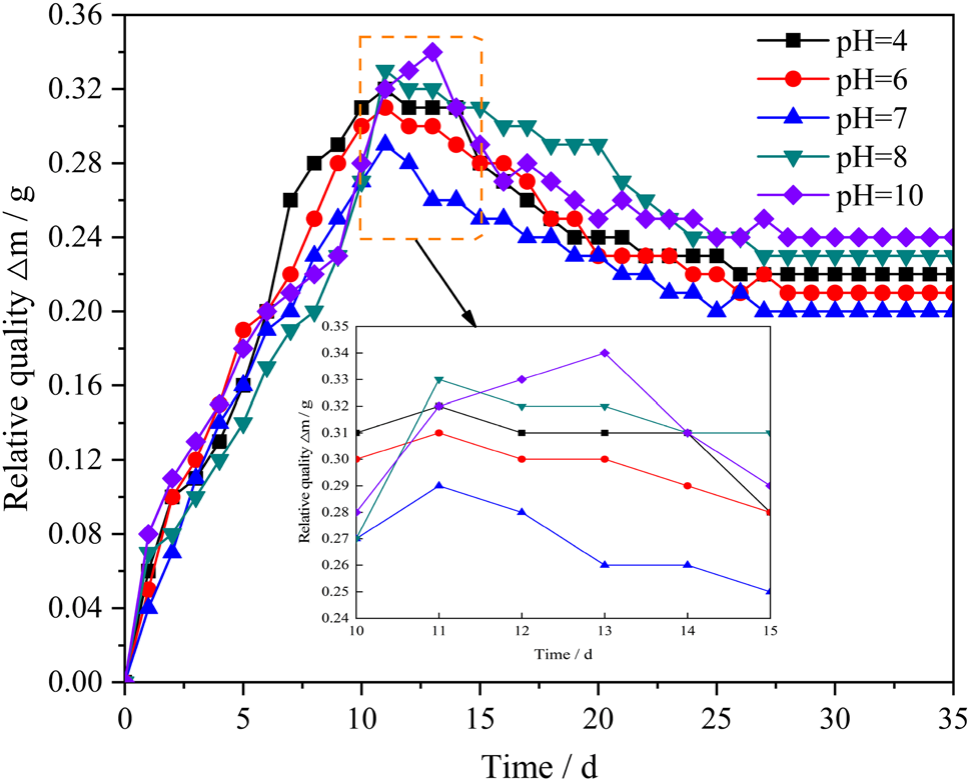
Relationship between action time of mine water and relative mass Δm of specimen.

Figure 5 shows the variation curve of the relative mass of the specimen with the soaking time for different pH values.

As shown in Fig. 5, the relative mass Δm of the specimens in different mine water solutions exhibits different changes as the soaking time is extended:

1. In various mine water solutions, the relative mass of the specimen first increased, then decreased, and finally stabilized as the soaking time was extended. This indicates that, during the initial soaking stage, the mine water solution penetrated the interior of the specimen through voids. However, because the mass of the soaked solution was significantly greater than that of the corrosive substance, the relative mass Δm of the specimen increased rapidly. As the soaking time gradually increased, the specimen gradually reached the saturation state^4^. Subsequently, the specimen changed from the initial water absorption state to the chemical corrosion state, and then changed from the initial water absorption state to the chemical corrosion state, and the chemical corrosion was dominant. This resulted in the interaction of some substances in the resin grout specimen with the mine water solution. Thus, voids and cracks were formed, which resulted in the gradual decrease of the relative mass Δm of the specimen.
2. In different mine water solutions, the relative mass Δm of the resin grout specimen essentially followed the same trend as the soaking time. Within 24 days of soaking, the relative mass Δm of the specimen changed more obviously, exhibiting a trend of first increasing and then decreasing. After 24 days, the relative mass Δm gradually stabilized and eventually remained constant.
3. When the resin grout specimen was soaked in acidic or alkaline mine water solution, as the acidity or alkalinity of the mine water solution became stronger, the change range of the specimen’s relative mass Δm increased. In the same period, the specimen’s relative mass Δm under acidic conditions was greater than that under alkaline conditions.

## UCS results and analysis

### Influence of mine water on deformation characteristics of resin grout

**Figure 6:**
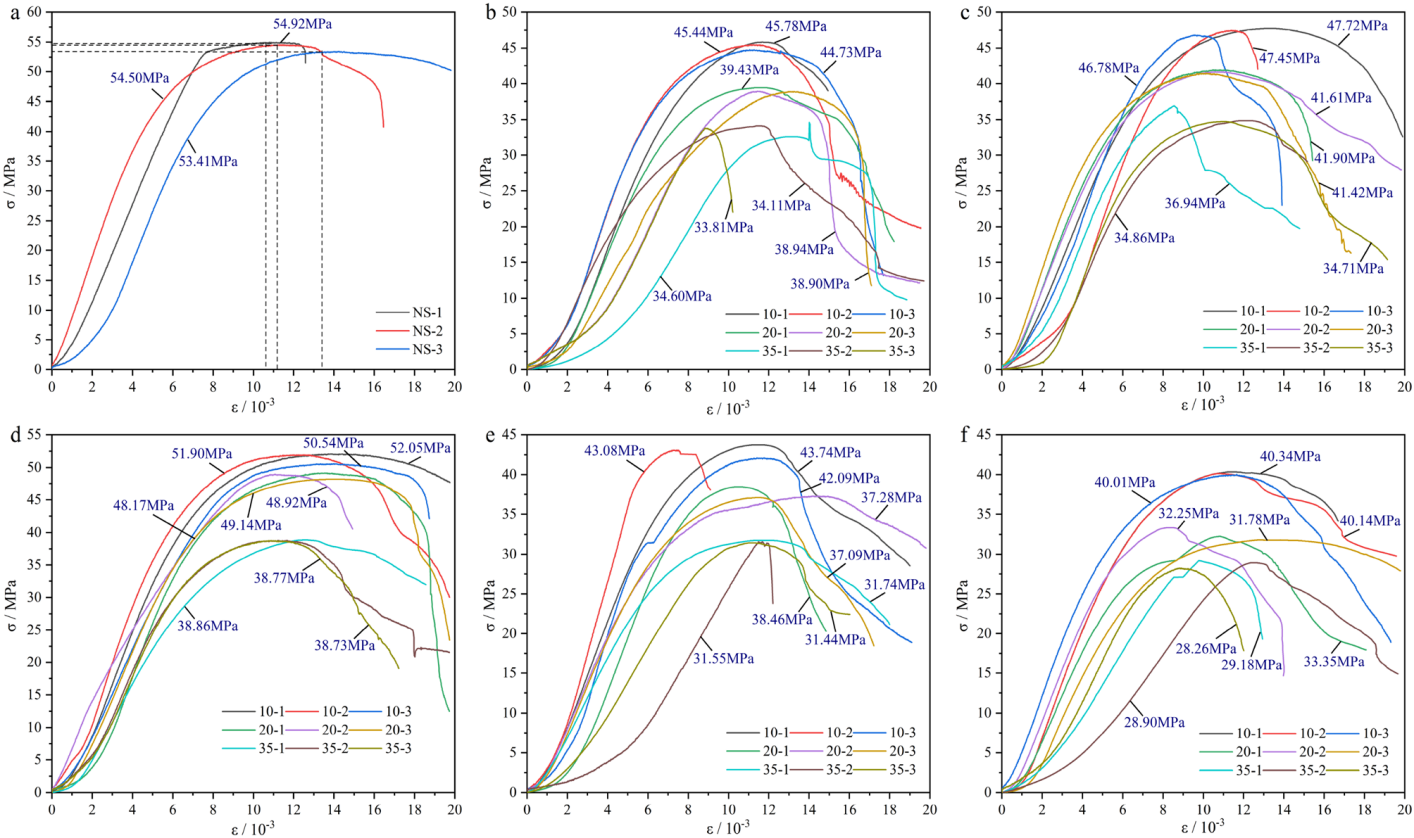
Stress–strain curves of uniaxial compression test specimens in different mine water environments: (**a**) NS; (**b**) pH 4; (**c**) pH 6; (**d**) pH 7; (**e**) pH 8; (**f**) pH 10.

In roadways and tunnels, the resin grout is exposed to various mine water environments and may erode. Accordingly, its mechanical properties will gradually deteriorate, affecting both the anchoring effect and anchoring quality. Therefore, it is important to investigate the changes in the mechanical strength of resin grout. By using a servo testing machine to conduct uniaxial compression tests on resin grout specimens, the stress–strain curves and uniaxial compression test results of the specimens under the same pH value but different soaking cycles were obtained as presented in Fig. 6 and Table 4. As shown in Fig. 6, the stress–strain curve of the resin grout can be approximately divided into four stages: the compaction stage, linear elastic stage, plastic stage, and failure stage.

1. *Compaction stage*. In the initial stage, the axial pressure of the specimen was small, the original micro cracks in the specimen gradually closed, the internal voids of the resin grout were compacted, early nonlinear deformation occurred, and the stress–strain curve exhibited an upward concave shape. As shown in Fig. 6, the upper concave section of the NS (Fig. 6a) lower specimen is shorter and exhibits less deformation than the specimen treated with mine water solution (Fig. 6b–f). Before the specimen entered the linear elastic stage, the compression stage of the specimen after the action of the mine water solution increased significantly. The main reason for this is that the mine water solution leads to the generation of micro cracks on the specimen’s surface and increases the initial crack density of the specimen. Therefore, the corroded specimen may exhibit a faster deformation rate during the compaction stage.
2. *Linear elastic stage*. As the axial pressure of the specimen increased, the resin grout specimen entered the linear elastic stage after the initial compaction stage. The stress–strain curve of this stage is approximately linear, and the slope of the straight line is the elastic modulus *E*. As shown in Fig. 6, compared with the NS (Fig. 6a), the elastic modulus of resin grout decreased to varying degrees under the action of the mine water solution (Fig. 6b–f). Under soaking with the same mine water solution, the elastic modulus of the specimen decreased as the soaking period increased. The main reason for this is the reaction between the mine water solution and the internal substances of the resin grout specimen, which leads to the destruction of the internal structure of the resin grout specimen, thus weakening the overall strength of the specimen. Consequently, the specimen soaked in the mine water solution may exhibit lower elastic modulus in the online elastic stage, and the elastic deformation ability of the specimen will also be affected.
3. *Plastic stage*. As the axial pressure of the specimen increased further, the resin grout specimen entered the plastic stage after the linear elastic stage. The specimen developed internal cracks, and these cracks connected and penetrated. However, the mine water solution accelerated the crack propagation of the resin grout, making the specimen more prone to plastic deformation. As shown in Fig. 6, the plastic stage of the resin grout specimens in NS (Fig. 6a) and after soaking in the mine water solution (Fig. 6b–f) is not obvious.
4. *Post-failure stage*. After the bearing capacity of the specimen reached the peak strength, its internal structure was destroyed. At this stage, the internal cracks of the specimen further developed and expanded, eventually leading to the large-scale damage of the internal structure of the specimen and the loss of bearing capacity. However, owing to the strong plasticity of resin grout, the fracture of the specimen was not accompanied by a crisp sound similar to the rock specimen, and only slight fracture occurred. As shown in Fig. 6, the stress of the resin grout specimen in the NS (Fig. 6a) decreased rapidly after failure, and the peak softening stage did not exist. In contrast, the specimen treated with the mine water solution (Fig. 6b–f) clearly exhibited different degrees of the peak softening stage.

**Table 4:** Statistics of uniaxial compression test results of resin grout specimens subjected to different mine water environments.

Solution type	Soaking time/d	Samples	UCS/MPa	Average UCS/MPa
NS	–	NS-1	54.92	54.27
		NS-2	54.50	
		NS-3	53.41	
pH = 4	10	10-1	45.44	45.32
		10-2	44.73	
		10-3	45.78	
	20	20-1	39.43	39.09
		20-2	38.94	
		20-3	38.90	
	35	35-1	34.60	34.17
		35-2	34.11	
		35-3	33.81	
pH = 6	10	10-1	45.72	47.32
		10-2	47.75	
		10-3	46.78	
	20	20-1	41.90	41.64
		20-2	41.61	
		20-3	41.42	
	35	35-1	36.94	35.50
		35-2	34.86	
		35-3	34.71	
pH = 7	10	10-1	52.05	51.50
		10-2	51.90	
		10-3	50.54	
	20	20-1	49.14	48.75
		20-2	48.92	
		20-3	48.17	
	35	35-1	38.86	38.79
		35-2	38.77	
		35-3	38.73	
pH = 8	10	10-1	43.74	42.97
		10-2	43.08	
		10-3	42.09	
	20	20-1	38.46	37.61
		20-2	37.28	
		20-3	37.09	
	35	35-1	31.74	31.58
		35-2	31.55	
		35-3	31.44	
pH = 10	10	10-1	40.34	40.16
		10-2	40.14	
		10-3	40.01	
	20	20-1	33.35	32.46
		20-2	32.25	
		20-3	31.78	
	35	35-1	29.18	28.78
		35-2	28.90	
		35-3	28.26	

### Effect of mine water on strength characteristics of resin grout

**Figure 7:**
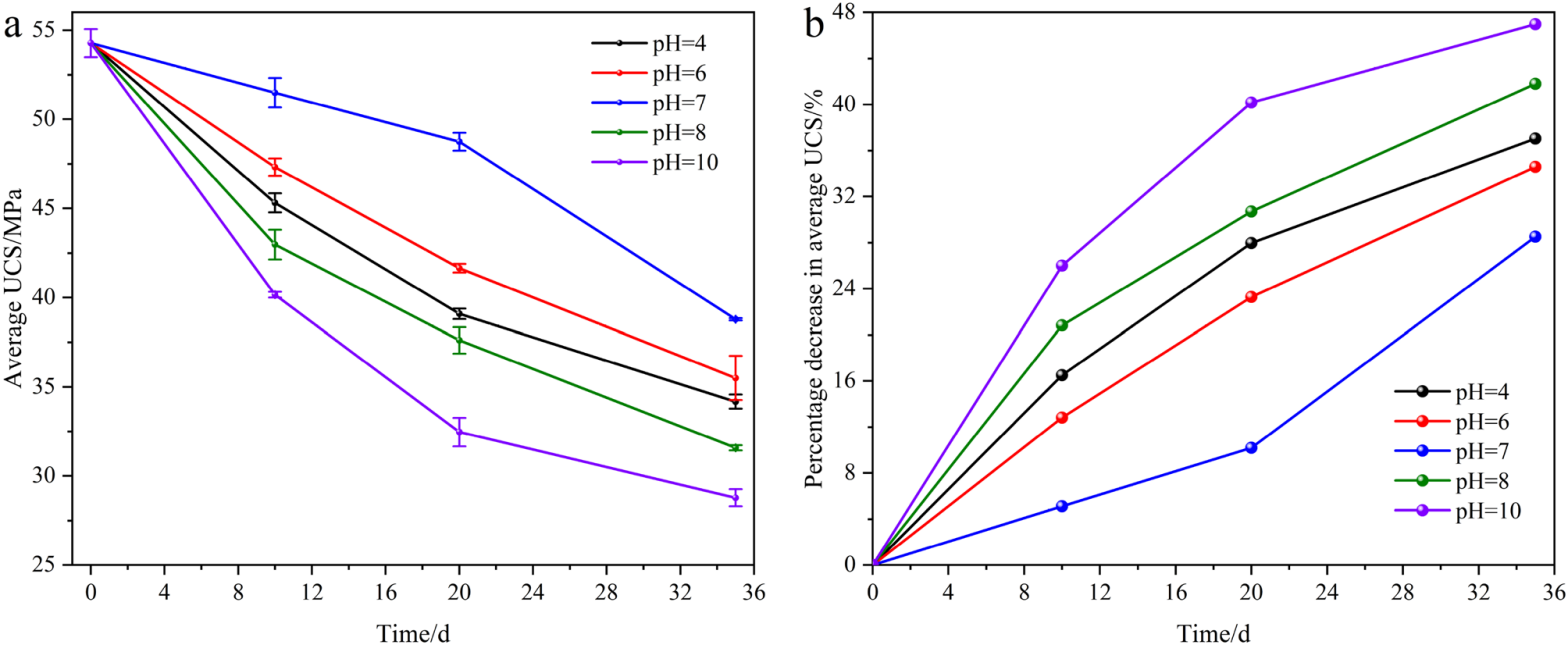
Variation of average UCS of resin grout under different soaking times: (**a**) average UCS; (**b**) percentage decrease in average UCS.

Figure 7 shows the average UCS variation and percentage decrease in the average UCS of the resin grout specimens soaked in mine water solution with different pH values for different time periods.

From Fig. 7, the following observations can be made:

1. After soaking the resin grout specimens in different mine water solutions for 10 d, the average UCS of the specimens was significantly lower than that of the specimens in the NS. After soaking at pH 4, 6, 7, 8, and 10 for 10 d, the average UCS of the specimens was 45.32 MPa, 47.32 MPa, 51.50 MPa, 42.97 MPa, and 40.16 MPa, respectively; compared with the NS (54.27 MPa) specimens, the strength decreased by 16.49%, 12.81%, 5.10%, 20.82%, and 26.00%, respectively. The main reason for this is that different mine water solutions destroyed the molecular chain of the polymer in the resin grout, reduced the molecular weight of the polymer, and thereby severely affected the resin grout by decreasing its strength.
2. After soaking the resin grout specimens in different mine water solutions for 20 d, the average UCS of the specimens was significantly lower than that of the specimens in the NS. After soaking at pH 4, 6, 7, 8, and 10 for 20 d, the average UCS of the specimens was 39.09 MPa, 41.64 MPa, 48.75 MPa, 37.61 MPa, and 32.46 MPa, respectively; compared with the NS (54.27 MPa) specimens, the strength decreased by 27.97%, 23.27%, 10.17%, 30.70%, and 40.19%, respectively. The above analysis also reveals that the specimen strength decreased further as the soaking time was extended in different mine water environments.
3. After soaking the resin grout specimens in different mine water solutions for 35 d, the average UCS of the specimens was more obvious than that of the specimens in the NS and those in the first two test cycles. After soaking at pH 4, 6, 7, 8, and 10 for 35 d, the average compressive strength of the specimens was 34.17 MPa, 35.50 MPa, 38.79 MPa, 31.58 MPa, and 28.78 MPa, respectively; compared with the NS (54.27 MPa) specimens, the strength decreased by 37.04%, 34.59%, 28.52%, 41.81%, and 46.97%, respectively. The above analysis also reveals that, after soaking in different mine water solutions for 35 d, the average UCS of the specimens was lower than that after soaking for 10 days and 20 d. Additionally, when soaking in the pH 10 solution for 35 d, the compressive strength was reduced by approximately half compared with that of the specimens in the NS.
4. With the other conditions being the same, when the resin grout specimen was soaked in an acidic, neutral, or alkaline mine water solution for different test cycles, the reduction of compressive strength in the alkaline mine water solution was greater than that in an acidic or neutral solution. The main reason for this is that the dolomite powder, light calcium, and other substances inside the resin and the ester bond of the polyester resin in the unsaturated polyester resin interacted with the H^+^ in the acidic mine water solution, which made the solution weakly alkaline. In the alkaline environment, the ester bond of the polyester resin in the unsaturated polyester resin was hydrolyzed by the action of alkali, and exhibited weak alkalinity after hydrolysis owing to the continuous weakening effect of various mine water solutions on the specimen as a result of the overall weak alkalinity of the solution after interaction with the specimen.
5. With the other conditions being the same, the specimen was soaked in different acidic and alkaline mine water solutions in the same test cycle, and the peak strength was compared with that of the average NS. As the acidity and alkalinity of the solution became stronger, the peak strength of the corresponding specimen decreased.

### Effect of mine water on elastic modulus and Poisson’s ratio of specimens

**Figure 8:**
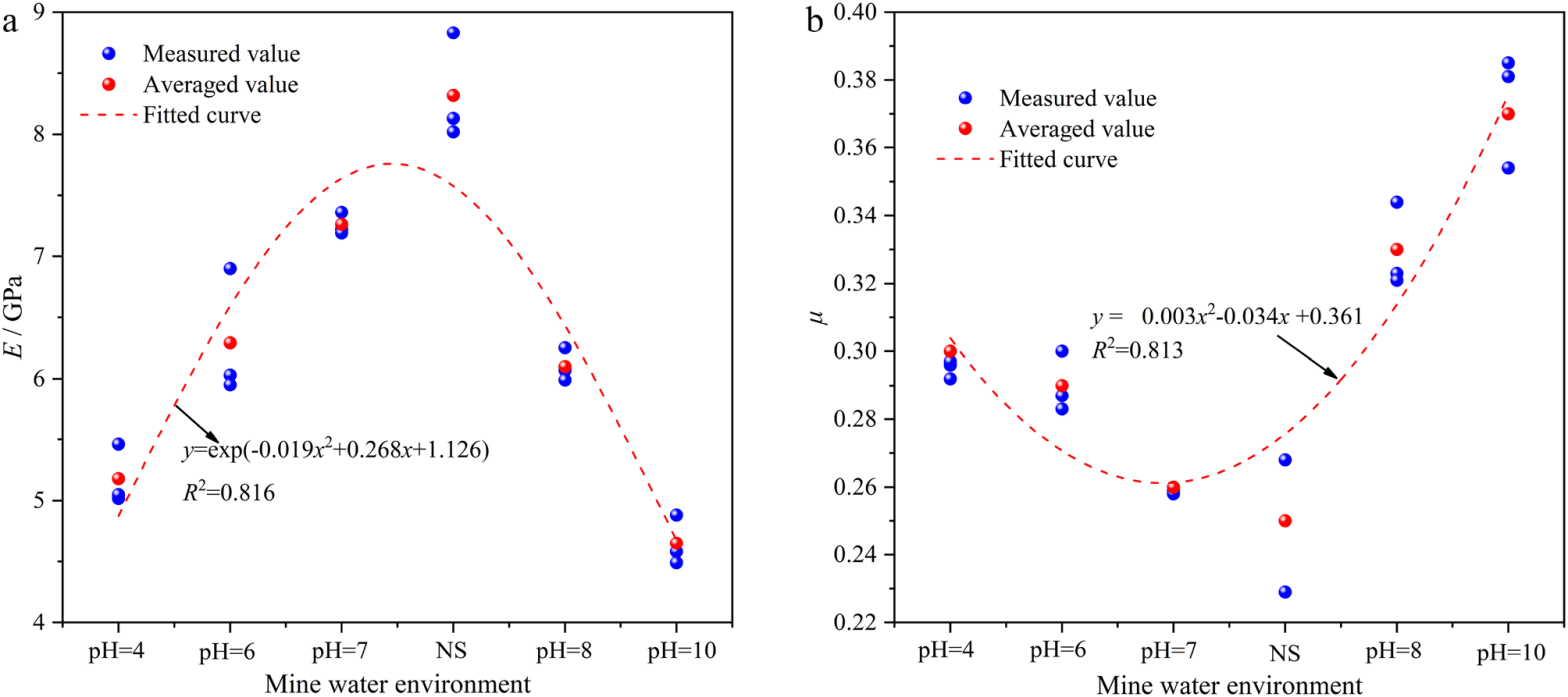
Effect of mine water corrosion on *E* and *μ* of resin grout: (**a**) *E*; (**b**) *μ*.

The elastic modulus and Poisson’s ratio are important mechanical properties reflecting the mechanical behavior of resin grout. The elastic modulus and Poisson’s ratio are expressed by *E* and *μ*, respectively. The resin grout specimens were soaked in mine water solution with different pH values for three test cycles, namely 10 d, 20 d, and 35 d; the *E* and *μ* values in each cycle were similar. Owing to space limitations, only the specimen soaked in different mine water solutions for 35 d is considered as an example, and the variations in the resin grout specimen’s *E* and *μ* values are shown in Fig. 8.

As shown in Fig. 8a, after soaking the specimens in different mine water solutions for 35 d, the average *E* of the specimens treated with the mine water solution was lower than that of the specimens in the NS. As the acidity and alkalinity of the mine water solution increased, the *E* of the resin grout gradually decreased. From pH 4 to NS to pH 10, *E* generally first increased rapidly and then decreased rapidly. The average *E* exhibited an approximate exponential relationship with the mine water solutions. After soaking for 35 d at pH 4, 6, 7, 8, and 10, the average *E* was 5.58 GPa, 6.29 GPa, 7.26 GPa, 6.10 GPa, and 4.65 GPa, respectively; compared with the NS (8.32 GPa) specimen, the average *E* decreased by 32.93%, 24.40%, 12.74%, 26.68%, and 44.11% respectively.

The above analysis reveals that the *E* of the resin grout changed in the mine water environment, indicating that the specimens interacted with the mine water solution. The degree of *E* reduction in the alkaline mine water solution is greater than that in the acidic mine water solution. Moreover, as the acidity and alkalinity became stronger, the degree of *E* degradation increased. The main reason for this is the interaction of the resin grout in the mine water environment, which caused the macromolecular chains in the resin to break, thereby promoting the swelling of the resin and destroying the interface between the resin and the filler. The rapid solidification in the resin grout led to temperature differences, resulting in the appearance of various internal microcracks, which further expanded with soaking in mine water. Therefore, the *E* of the resin grout specimens treated with mine water solution was lower than that of specimens in the NS.

As shown in Fig. 8b, after soaking the resin grout specimens in different mine water solutions for 35 d, the average *μ* of the specimens was greater than that of the specimens in the NS. Furthermore, as the acidity or alkalinity of the mine water solutions increased, the *μ* of the resin grout generally first decreased rapidly and then increased rapidly from pH 4 to NS to pH 10. The average *μ* exhibited an approximate quadratic relationship with the mine water solutions. After soaking at pH 4, 6, 7, 8, and 10 for 35 d, the average *μ* was 0.30, 0.29, 0.26, 0.33, and 0.37, respectively; compared with the NS (0.25) specimens, the average *μ* increased by 20%, 16%, 4%, 32%, and 48%, respectively, and *μ* in the alkaline mine water environment was greater than that in the acidic environment. The main reason for this is the interaction between the H^+^ ions and the substances inside the specimen under an acidic mine water environment, and the hydrolysis of polyester resin in the unsaturated polyester resin under acidic conditions. Under alkaline conditions, the substances inside the specimen could not dissolve easily, and only a small portion underwent hydrolysis reaction. Eventually, however, all types of mine water tended to be weakly alkaline, owing to the continuous weakening effect of resin grout. In 1996, Christensen^35^ conducted many tests to investigate the effects of various factors on *μ*. The experimental results revealed that the influence of pressure and temperature on *μ* is very small, whereas the change of the chemical composition greatly affects *μ*. For example, Poisson’s ratio increased after the Mg in pyroxene and olivine was replaced by Fe. When the resin grout was soaked in different mine water solutions, various substances in the specimen interacted with the mine water solution, resulting in changes in the specimen’s material composition and, therefore, changes in *μ*.

## Analysis of corrosion mechanism of resin grout in mine water solution

### Definition of damage parameters and their influence on compressive strength of resin grout

The selection of appropriate damage variables is important for investigating the damage effect of a mine water solution on resin grout specimens. There are many damage variable definitions, and appropriate state variables are commonly selected as the damage variables for measuring the damage of specimens both from macro and micro viewpoints^4,36^. From a macro perspective, the yield stress, tensile strength, density, elastic constant, and acoustic emission strength of the specimens can be selected. From a micro perspective, the number, length, area, and volume of voids in the specimen can be selected. As discussed in Section "Change of porosity of specimens before and after soaking", after the resin grout specimen was soaked in the mine water solution, the specimen’s porosity increased, and the corresponding void structure changed. Therefore, the porosity damage variable can reflect the degree of corrosion damage induced by the mine water solution to the resin grout; the damage parameter *D* is expressed as follows:

4 $$ D = \frac{{n_{t} - n_{0} }}{{1 - n_{0} }} $$

**Figure 9:**
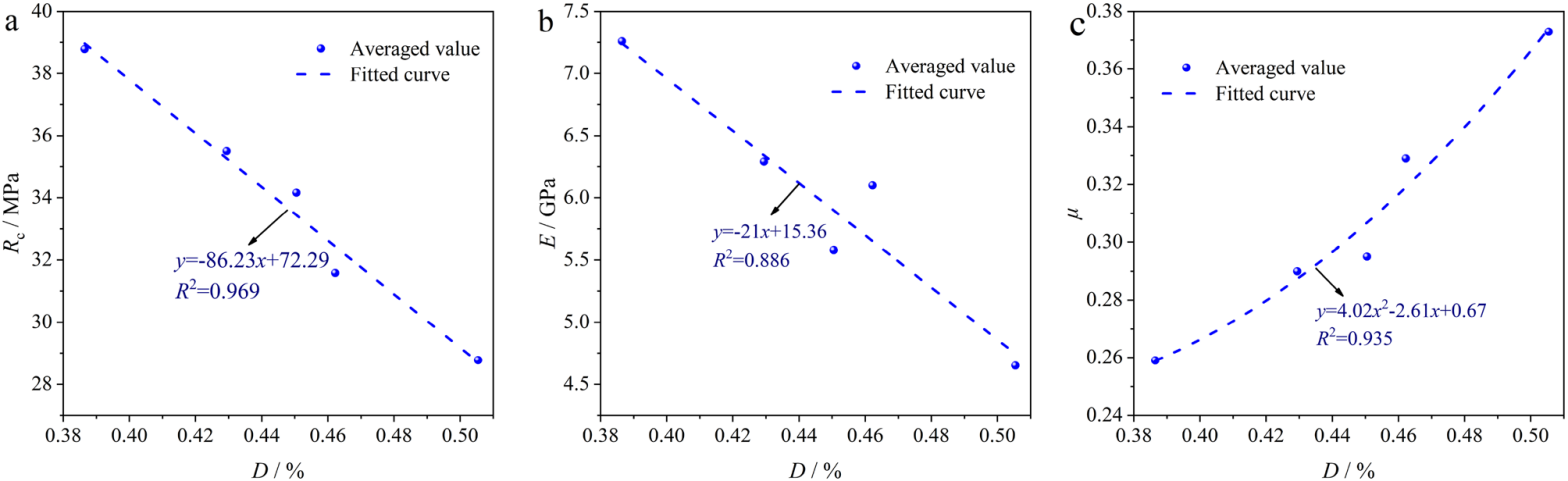
Relationships of mechanical properties with damage parameter of resin grout: (**a**) uniaxial compressive strength; (**b**) elastic modulus; (**c**) Poisson’s ratio.

Combined with the test data in Section "Change of porosity of specimens before and after soaking", the corresponding damage parameter *D* of the resin grout after corrosion in different mine water solutions and soaking times can be obtained by Eq. (4). The resin grout specimens were soaked in the mine water solution with different pH values for three test cycles (10 d, 20 d, and 35), and similar specimen damage parameters were used in each cycle. Owing to space limitations, only the specimen soaked in different mine water solutions for 35 d is considered as an example. The relationships of the damage parameter *D* with *R*_c_, *E*, and *μ* of the resin grout are analyzed in Fig. 9.

From the regression analysis in Fig. 9, the following expression can be obtained:

5 $$ \left\{ \begin{gathered} R_{{\text{c}}} = - 86.23D + 72.29(R^{2} = 0.969) \hfill \\ E = - 21D + 15.36(R^{2} = 0.886) \hfill \\ \mu = 4.02D^{2} - 2.61D + 0.67(R^{2} = 0.935) \hfill \\ \end{gathered} \right. $$

From Fig. 9 and the related regression Eq. (5), it is concluded that the *R*_c_ and *E* of resin grout exhibited decreasing trends as the damage parameter *D* increased, whereas *μ* exhibited an increasing trend as the damage parameter *D* increased. Specifically, *R*_c_ and *E* decreased linearly whereas *μ* increased quadratically with the damage parameter *D*. This further demonstrates that the corrosion effects of different acidic or basic mine water solutions on the resin grout in the anchoring system gradually accumulate as the corrosion cycle is extended. Eventually, this damage accumulation leads to the deterioration of the relevant mechanical properties of the resin grout specimen.

### Type of action of mine water and resin grout

The change of the macroscopic mechanical properties of resin grout under the action of mine water solution results from the change of the resin grout internal microstructure. When different mine water interacts with resin grout, the composition, size, shape, and mineral particles inside the resin grout change. Moreover, different mine water solutions interact with the resin grout to different degrees. The results obtained through the soaking test and uniaxial compression test reveal that the resin grout performance was markedly affected by the mine water solution. In the early stage, the quality of resin grout before and after corrosion, and the change of the pH value of the mine water solution during corrosion, were measured. On this basis, the mechanism and characteristics of the interaction between the mine water solution and the resin grout were analyzed in depth. The conclusions drawn from the analysis are as follows:

1. When the resin grout was soaked in acidic mine water solution, the pH value of the mine water solution increased and gradually tended to stabilize over time. The main reason for this is the hydrolysis of the ester bond in the polyester resin under acidic conditions and the interaction of H^+^ ions in the solution of the fillers (dolomite powder and light calcium) and other substances in the resin grout under acidic conditions, which broke the macromolecular chain of the resin, destroyed the bonding of the interface between the resin and the filler, and reduced the resin grout strength. The acidic mine water reacted with the resin grout as follows:6
  7 $$ {\text{CaMg}}\left( {{\text{CO}}_{3} } \right)_{2} + \, 4{\text{ H}}^{ + } \to {\text{Ca}}^{2 + } + {\text{Mg}}^{2 + } + 2{\text{H}}_{2} {\text{O }} + \, 2{\text{CO}}_{2} \uparrow $$
  8 $$ {\text{CaCO}}_{3} + \, 2{\text{ H}}^{ + } \to {\text{Ca}}^{2 + } + {\text{ H}}_{2} {\text{O}} + {\text{ CO}}_{2} \uparrow $$
  9 $$ {\text{CaO }} + \, 2{\text{ H}}^{ + } \to {\text{Ca}}^{2 + } + {\text{ H}}_{2} {\text{O}} $$
2. When the resin grout was soaked in a neutral mine water solution, the pH value of the solution slightly increased and then tended to be stable. This was mainly caused by the exchange of H^+^ ions in the solution with the alkaline cations of some substances in the resin grout specimen under neutral conditions, which resulted in the pH value of the solution tending toward weak alkalinity. Additionally, some substances in the resin grout underwent slight hydrolysis reactions with the mine water solution. As the dissolution process progressed, the neutral mine water gradually transformed into weakly alkaline mine water, which led to a decrease in the resin grout strength.
  10 $$ {\text{CaCO}}_{3} + {\text{CO}}_{2} + {\text{H}}_{2} {\text{O}} \to {\text{Ca}}({\text{HCO}}_{3} )_{2} $$
  11 $$ {\text{CaO}} + {\text{H}}_{2} {\text{O}} \to {\text{Ca}}({\text{OH}})_{2} $$
  12 $$ {\text{SiO}}_{2} + 2{\text{H}}_{2} {\text{O}} \to {\text{H}}_{4} {\text{SiO}}_{4} $$
3. When the resin grout was soaked in an alkaline solution, the overall pH value of the solution decreased, and eventually tended toward a stable state. This was mainly caused by the quartz powder in the resin grout, which can easily react under alkaline conditions, and the ester bond in the polyester resin in the unsaturated polyester resin, which can easily hydrolyze under alkaline conditions. Additionally, because the fillers (dolomite powder and light calcium) in the resin grout cannot dissolve easily under alkaline conditions, only a few hydrolysis reactions occurred. This series of reactions changed the resin grout internal structure, destroyed the bond between the resin and the filler, and reduced the resin grout compressive strength.13
  14 $$ {\text{SiO}}_{2} + 2{\text{OH}}^{ - } \to {\text{SiO}}_{3}^{2 - } + {\text{H}}_{2} {\text{O}} $$
  15 $$ CaMg\left( {CO_{3} } \right)_{2} + 2H_{2} O + 2CO_{2} \to Ca^{2 + } + Mg^{2 + } + 4HCO_{3}^{ - } $$

### Microstructure change of resin grout before and after mine water action

**Figure 10:**
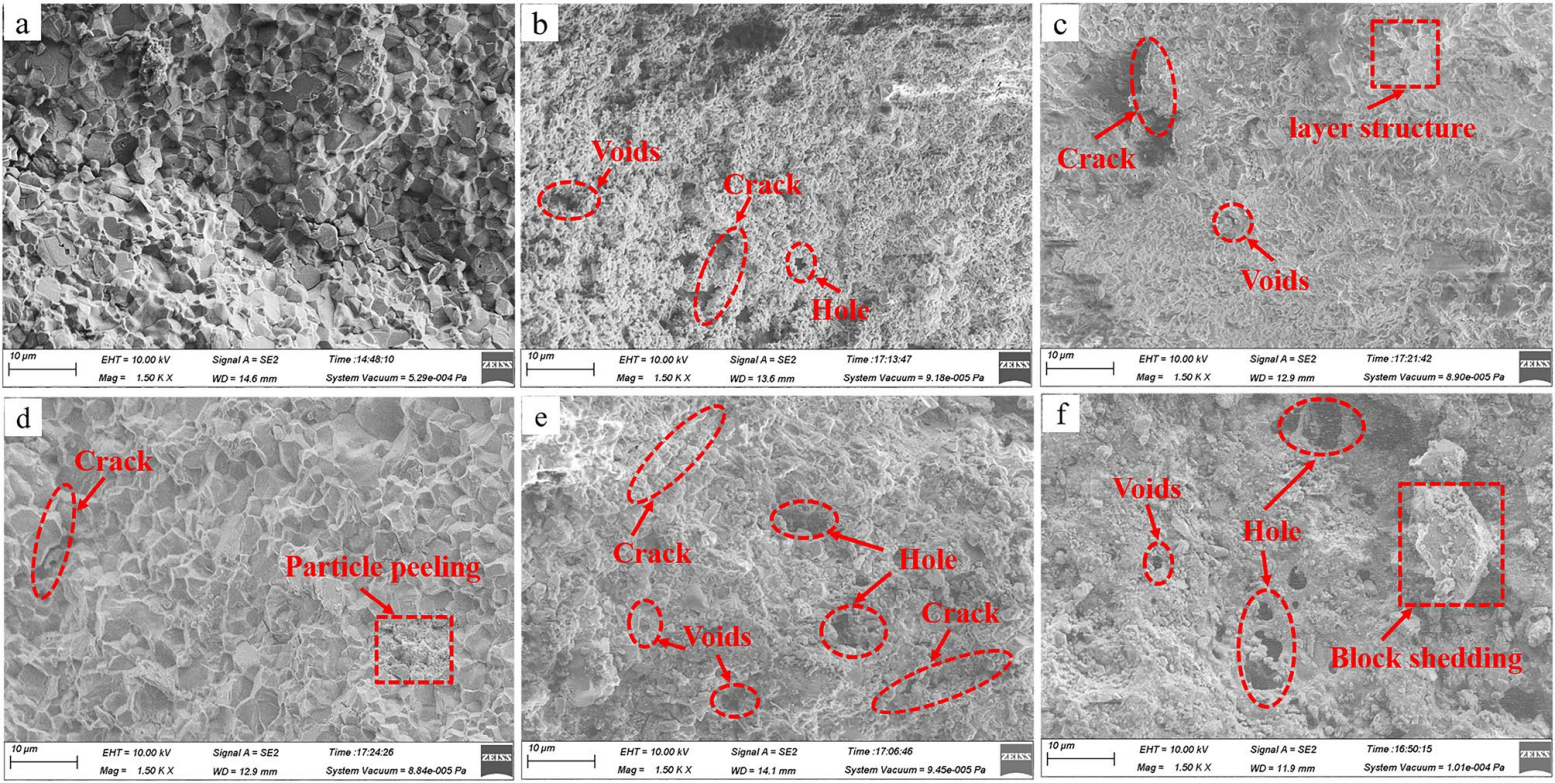
SEM images of resin grout soaked in different mine water solutions: (**a**) NS; (**b**) pH 4; (**c**) pH 6; (**d**) pH 7; (**e**) pH 8; (**f**) pH 10.

The interaction between different mine water solutions and the resin grout changed the resin grout microstructure to different degrees. Therefore, the specimens soaked in different mine water solutions were observed through SEM with a magnification of 1500 times to obtain images of the specimens’ microstructure before and after the action of different mine water solutions, as shown in Fig. 10.

As shown in Fig. 10, the internal particles of resin grout in the NS are mostly irregular blocks; the particles mainly have surface-to-surface contact, the inter-particle voids are small, and the overall structure is relatively dense (Fig. 10a). After the specimen was subjected to the action of acidic mine water solution, a large amount of its internal cementing material dissolved under the action of acidic mine water, and the block crystal decomposed. The original block particles no longer existed, and were replaced by layered and fine cluster particles. The large number of broken particles increased the voids and cracks. Additionally, as the acidity of the acidic mine water increased, the voids and cracks of the specimen significantly increased, and holes even appeared locally (Fig. 10b, c). After the specimen had been subjected to the action of neutral mine water solution, the internal cementing material was hydrolyzed under the action of neutral mine water, and the original massive particles dissolved into flake particles. The particle size of the mineral particles changed markedly, and voids and cracks appeared locally between the crystals. Moreover, the phenomenon of the edges and corners of some crystal particles eroding under the action of mine water was observed (Fig. 10d).

After the specimen was subjected to the action of alkaline mine water solution, the internal cementing material was hydrolyzed under the action of alkaline mine water, which destroyed the specimen’s original stable chemical bond, and the hydrolysis product attached to the crystal surface with a granular form. Voids and cracks appeared between the crystals, and large holes appeared locally. With the dissolution of the cementing material and the disintegration of the mineral itself, the crystal partially fell off, and as the alkalinity of the alkaline mine water solution increased, the voids, cracks, and holes of the specimen increased significantly (Fig. 10e, f).

In summary, with the soaking of resin grout in different mine water solutions, the microstructure and material composition of the specimen itself changed, which is closely related to the change of its macroscopic mechanical properties. These changes include changes in mechanical properties such as the UCS and elastic modulus, and microscopic changes that mainly manifested as changes in the composition and structure of the resin grout specimen. The change of the mechanical properties of the resin grout specimen is the external manifestation of the change of its microstructure and composition, whereas the change of the microstructure and material composition of the specimen is the internal cause of the change of the specimen’s macro-mechanical properties.

## Discussion and recommendations

The results indicate that resin grout, acting as the bonding medium between the bolt/cable bolt and the surrounding rock, will undergo changes in its internal structure and material composition when encountering mine water on the roof of roadways and tunnels. This leads to the deterioration of the mechanical properties and anchoring quality, which affects the long-term stability of the anchoring system. Zheng et al.^18^ found that, when resin grout was placed in water, the mechanical properties and surface stability of the test blocks changed significantly. Li^26^ investigated the compressive strength of the resin grout in different alkaline water environments and different corrosion cycles, and found that the compressive strength exhibited a decreasing trend compared with the natural state. As the alkalinity of the solution increased, the deterioration of the compressive strength of the resin grout became more severe. The findings of this study confirm the deterioration of the mechanical properties of resin grout reported by Zheng and Li. However, Zheng and Li did not systematically analyze the influence of different mine water solutions on the mechanical properties of resin grout or the influence of the deterioration of resin grout on the stability of the anchoring system.

In different mine water environments, the mechanical properties of the resin grout deteriorated to varying degrees compared with the NS. Among all mechanical properties, the UCS of resin grout is crucial. The deterioration of compressive strength will inevitably cause the partial failure of the anchoring system. Therefore, when the roadway and tunnel are anchored in areas with different mine water environments, different countermeasures should be taken with consideration to the selected resin grout.

In roof water drenching areas (particularly acidic or alkaline mine water solution), the mechanical properties of unsaturated polyester resin grout severely deteriorate owing to corrosion caused by the mine water environment during the roadway and tunnel support process, resulting in the anchorage failure of the surrounding rock of the roadway and tunnel. Therefore, when roadways and tunnels pass through these areas during construction, bisphenol A unsaturated polyester resin grout should be used because it is resistant to acidic and alkaline solutions. In the molecular structure of this resin grout type, the ester group concentration that can easily be destroyed by hydrolysis is much smaller than that in the molecular structure of typical unsaturated polyester, and the ester group is protected by the space barrier of an adjacent methyl group and cannot be easily invaded by medium molecules. Thus, bisphenol A resin achieves a high retention ratio for the mechanical properties, in addition to having good appearance^37,38^. Moreover, the use of bisphenol A resin grout in acidic and alkaline mine water environments can ensure good anchoring and prevent problems such as roadway and tunnel roof collapse caused by poor anchoring.

## Conclusions

This study considered the effects of different mine water solutions and soaking times on the resin grout, used a series of soaking tests and uniaxial compression tests to investigate the changes in the solution’s pH value and uniaxial compressive strength, and analyzed the deterioration caused by the mine water solution to the resin grout’s mechanical properties. Furthermore, beneficial engineering application countermeasures are proposed. The following conclusions were drawn from this study:

1. The corrosion effect of mine water solution on resin grout has a certain dependence on time and obvious stages. On the basis of the amplitude of the pH change of the mine water solution, the entire process of interaction can be divided into three approximate stages: the rapid reaction stage (0–75 h), the slow reaction stage (75–275 h), and the stable stage (after 275 h). After a certain period, the different solutions of acidic or alkaline mine water gradually tended toward weak alkalinity.
2. The mechanical properties of resin grout exhibited different degrees of deterioration in different mine water solutions. Compared with the UCS and elastic modulus of resin grout in the NS, as the acidity and alkalinity of the mine water solution became stronger and the soaking time was extended, the mechanical properties of the resin grout deteriorated to varying degrees, and Poisson’s ratio increased.
3. On the basis of the correlation between the changes in the porosity of the specimens, and the deterioration degree of the mechanical properties during interaction between the resin grout and different mine water solutions, the damage parameter *D* was defined. The UCS of the resin grout and the damage parameter *D* decreased linearly, the elastic modulus *E* approximately followed an exponential function with the damage parameter *D*, and the Poisson's ratio *μ* approximately followed a quadratic function with the damage parameter *D*.
4. The interaction between different mine water solutions and the resin grout resulted in different microstructural changes. As the acidity and alkalinity of the solution became stronger, more voids, cracks, and holes appeared in the resin grout, resulting in changes in the resin grout’s macroscopic mechanical properties.
5. The water environment of geotechnical and underground engineering is complex and variable, and the corrosion of resin grout is an extremely complex process. Future work should investigate the corrosion mechanism of resin grout with consideration of the actual situation of the site and the influence of various factors (including ions, temperature, and water pressure). The effective use of resin grout in engineering can be ensured by adjusting the formula of resin grout, selecting a suitable resin grout, or implementing waterproofing measures.

## Data Availability

The datasets used and analyzed during the current study available from the first author on reasonable request.
